# A rare presentation of Wilson disease with normal levels of serum ceruloplasmin and copper and MODY: A case report

**DOI:** 10.1097/MD.0000000000043080

**Published:** 2025-07-04

**Authors:** Meihong Han, Zhen Yang

**Affiliations:** a Department of Infectious Diseases, Provincial Hospital Affiliated to Shandong First Medical University, Jinan, Shandong.

**Keywords:** case report, ceruloplasmin, genetic metabolic disease, MODY, Wilson disease

## Abstract

**Rationale:**

Wilson disease (WD) is an autosomal recessive disorder in which mutations in *ATP7B* lead to excessive copper deposition in the liver, brain, eyes, kidneys, and other organs. Patients with WD can present with hepatic dysfunction, neurological symptoms, rare hemolytic anemia, and renal tubular acidosis.

**Patient concerns:**

A 27-year-old female patient with liver cirrhosis and maturity-onset diabetes of the young was found to have normal serum ceruloplasmin (25.9, 20–60 mg/dL) and copper levels (17.81, 12.6–23.6 μmol/L).

**Diagnoses:**

Liver biopsy revealed severe lobular hepatitis (multiple lobular/large necroses), early cirrhosis with a small amount of copper deposits, hepatocyte lipidosis, balloon-like changes, Mallory bodies, and glycogenated hepatocyte nuclei. Genetic analysis of *ATP7B* exon found 2 variants (c.2333G>T, p. Arg778Leu) in exon 8 and (c.3209C>G, p. Pro1070Arg) in exon 14. Based on the available results, the Leipzig score was 7 and the diagnosis of WD can be confirmed. A late Kayser–Fleischer corneal rings test came back positive.

**Interventions:**

The patient’s clinical management included d-penicillamine and insulin.

**Outcomes:**

At approximately 6 months of follow-up, the patient’s liver function was well controlled and the cirrhosis did not progress or have decompensated events.

**Lessons:**

Although WD is treatable, its early detection is challenging. Genetic testing and liver pathology are sometimes necessary for the diagnosis of WD. This case underscores the consideration of WD in the differential diagnosis of patients with liver cirrhosis. Normal ceruloplasmin levels do not rule out WD.

## 1. Introduction

Wilson disease (WD) is an autosomal recessive disorder caused by mutations in *ATP7B*, which encodes a copper-transporting P-type ATPase that promotes copper excretion from bile. Excessive Cu is deposited in the liver, brain, kidneys, and other organs, leading to damage in patients with WD.^[1]^

However, the diagnosis of WD can be challenging. Patients may have an insidious onset or nonspecific symptoms. Diagnostic tests including serum ceruloplasmin (Cp) levels may yield false-negative results. The typical features of WD (low serum copper, low Cp, and high urinary copper levels) are incomplete or absent in 3% of patients with WD confirmed by genetic testing and present in 16% of healthy heterozygous carriers.^[2]^ This report presents the case of a patient with WD who did not show increased serum ceruloplasmin or copper levels.

Maturity-onset diabetes of the young (MODY) is an inherited diabetes mellitus classically presenting in adolescence or young adults before the age of 25 years. Diagnosis of MODY is insufficient and is frequently misdiagnosed as type 1 diabetes or type 2 diabetes. The relationship between these 2 genetic disorders (WD and MODY) is rarely reported.

## 2. Case report

A 27-year-old female patient with a history of diabetes was admitted to the hospital with liver cirrhosis of unknown etiology. Twenty years prior to her presentation, the patient had abnormal liver biomarker levels during an upper respiratory tract infection that could not be attributed to immunological, copper or iron metabolic, or viral causes. Approximately 4 years prior to her presentation, the patient was diagnosed with liver cirrhosis and had been using Chinese herbs for approximately 2 years. The patient denied anorexia, fatigue, ventosity, lipophobia, weight loss, dyskinesia, involuntary movements, or cognitive impairment. The patient had no history of liver disease or alcohol consumption.

The patient had a normal body mass index without liver palm or spider nevus. She tested negative for diabetic autoantibodies (including glutamic acid decarboxylase antibodies, islet cell antibodies, and insulin autoantibodies). Tests for hepatitis A, B, C, and E antibodies and autoimmune hepatitis antibodies were also negative. The patient’s iron metabolism level was normal. She had normal ceruloplasmin and serum copper levels, but the 24-hour urine copper and serum free copper levels were elevated (Tables 1 and 2).

**Table 1:** Copper metabolism.

	Levels	Reference range
Ceruloplasmin	25.9 mg/dL	20–60 mg/dL
Serum copper level	17.81 μmol/L	12.6–23.6 μmol/L
Urinary copper/24 h	308.217 μg/L	0–160 μg/L
Serum free copper level	315 μg/L	<150 μg/L

**Table 2:** Liver injury factor screening.

	Levels	Reference range	Results
Serum Fe level	20.47 μmol/L	7.8–32.2 μmol/L	Normal
Transferrin saturation (TS)	30.91%	20%–55%	Normal
HBsAb	129.52 mIU/mL	<10 mIU/mL	Positive
Anti-HAV-IgM	0.04 S/CO	<1.0 S/CO	Negative
Anti-HEV-IgM	0.06 S/CO	<1.0 S/CO	Negative
HIV-Ag/Ab	0.24 S/CO	<1.0 S/CO	Negative
TPAb	0.06 S/CO	<1.0 S/CO	Negative
ANA	<1:100	<1:100	Negative
AMA	0.4 AU	0–20 AU	Negative
LC-1	3.00	0–15	Negative
SLA/LP	7.00	0–15	Negative
LKM1	3.00	0–15	Negative
Sp100	6.00	0–15	Negative
Gp210	6.00	0–15	Negative
IgG	19.8 g/L	8.6–17.4 g/L	Elevated
IgM	1.43 g/L	0.5–2.8 g/L	Normal
IgG4	697 mg/L	30–1350 mg/L	Normal
COR8:00	218 nmol/L	166–507 nmol/L	Normal
ACTH8:00	17.4 pg/mL	7.2–63.3 pg/mL	Normal
FT3	4.14 pmol/L	2.43–6.01 pmol/L	Normal
FT4	10.35 pmol/L	9.01–19.05 pmol/L	Normal
TSH	0.9931 μIU/mL	0.35–4.94 μIU/mL	Normal
Anti-TG	12.2 IU/mL	0–115 IU/mL	Negative
Anti-TPO	32.6 IU/mL	0–34 IU/mL	Negative
TRAb	<0.8 IU/L	0–1.22 IU/L	Negative

ACTH = adreno-cortico-tropic-hormone, AMA = anti-mitochondrial antibody, ANA = antinuclear antibody, Anti-HAV-IgM = IgM anti-HAV antibody, Anti-HEV-IgM = IgM anti-HEV antibody, Anti-TG = thyroglobulin antibody, Anti-TPO = anti-thyroid peroxidase antibody, COR = cortisol, FT3 = free triiodothyronine, FT4 = free thyroxine, Gp210 = autoantibody to gp210, HAV = hepatitis A virus, HBsAb = hepatitis B virus s antibody, HEV = hepatitis E virus, HIV = human immunodeficiency virus, IgG = immunoglobulin G, IgG4 = immunoglobulin G4, IgM = immunoglobulin M, LC-1 = autoantibody to LC-1, LKM1 = autoantibody to liver kidney microsomes-1, SLA/LP = autoantibody to soluble liver antigen/liver pancreas, Sp100 = autoantibody to sp100, TPAb = treponema pallidum antibody, TRAb = thyrotropin receptor antibody, TSH = thyroid-stimulating hormone.

**Figure 1.**
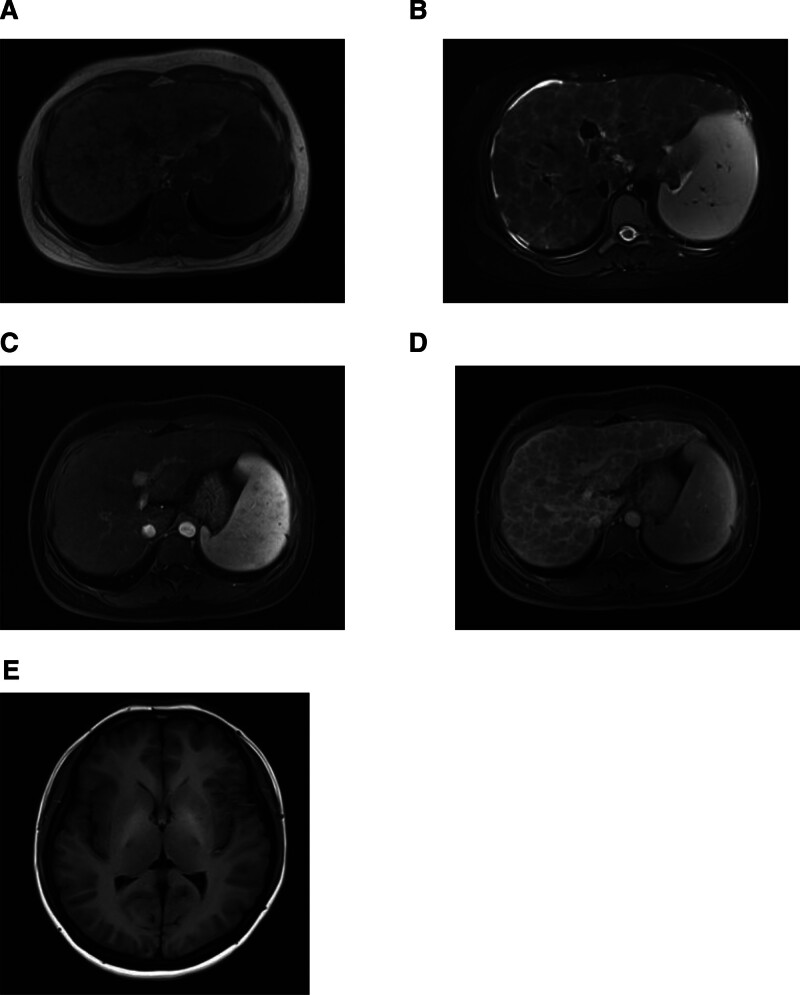
Hepatic and cerebral magnetic resonance imaging. Diffuse nodules appear distributed in the hepatic parenchyma, separated by fibrous septa, with an isointense signal on T1-weighted imaging (A), and a hyperintense signal on T2-weighted imaging (B). The nodules are not enhanced during the arterial phase (C). The fibrous septum is more pronounced in the venous phase (D). Bilateral pallidus abnormalities are observed on T1 head imaging (E).

The patient had elevated glutamyltranspeptidase (148 U/L, range: 7–45 U/L) and alkaline phosphatase (105 U/L, range: 35–100 U/L) levels and normal aspartate aminotransferase (30 U/L, range: 13–35 U/L), alanine aminotransferase (40 U/L, range: 7–40 U/L), and bilirubin levels (20.4 μmol/L, range: 0–21 μmol/L). A honeycomb pattern was observed on liver magnetic resonance imaging (Fig. 1A–D). The common magnetic resonance imaging manifestations are symmetrical bilateral lenticular nuclei with hypointensities on T1 weighted imaging and hyperintensities on T2 weighted imaging.^[3]^ T1-weighted axial magnetic resonance imaging reveals bilateral hyperintensities in the globus pallidus (Fig. 1E), although the patient remained asymptomatic neurologically.

Liver biopsy revealed occasional copper deposition, hepatocyte steatosis, balloon-like degeneration, Mallory bodies, and major glycogenation of hepatic nuclei. The hepatic parenchyma was irregularly separated by collapsed zones of bridging necrosis and multilobular necrosis with moderate mononuclear cell infiltration and few plasma cells in the necrotic zone. Reactive proliferation was significant within the fine bile duct with limited hepatocyte differentiation. Rhodanine staining revealed the presence of positive particles in hepatocytes. Regeneration nodules were also observed. Marked nuclear variability with glycogen vacuolization in the nuclei was observed. Some hepatocytes showed bulla steatosis and balloon-like degeneration. Mallory bodies have also been observed, suggesting the possibility of WD.^[4]^

**Figure 2.**
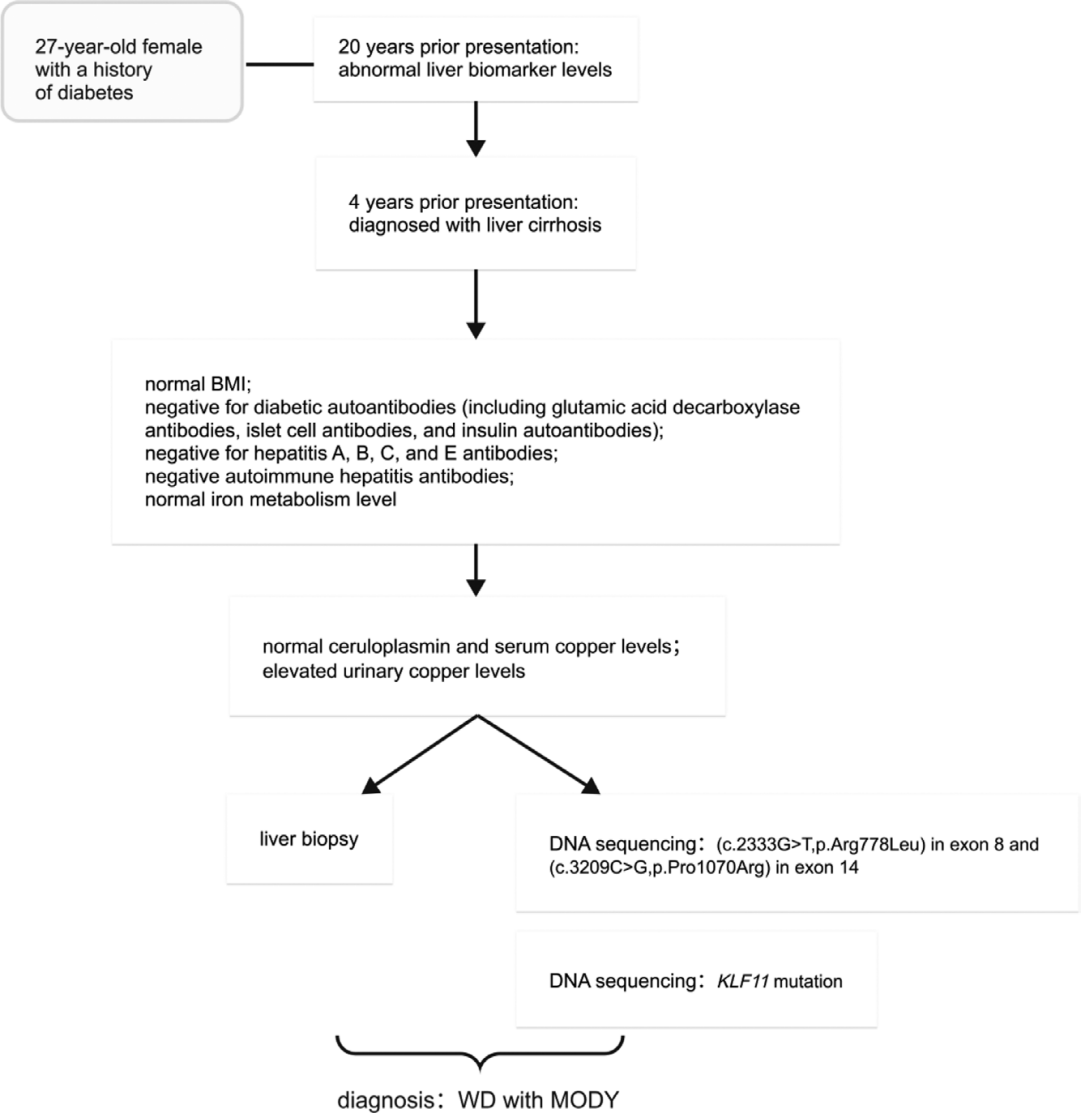
Flow chart for patients history.

Direct DNA sequencing of *ATP7B* exons was performed because of the patient’s atypical presentation. One pathogenic heterozygous mutation (c.2333G>T, p. Arg778Leu) in exon 8 and another pathogenic heterozygous mutation (c.3209C>G, p. Pro1070Arg) in exon 14 were identified. The patient’s parents also had mutations in *ATP7B* exons. The mother had 1 heterozygous mutation (c.2333G>T, p. Arg778Leu) in exon 8 and the father had 1 heterozygous mutation (c.3209C>G, p. Pro1070Arg) in exon 14. Therefore, the patient had inherited 1 mutation from each parent. The patient was also found to have a likely pathogenic variant of the Krüppel-like factor 11 (*KLF11*) gene, associated with MODY^[5]^ (the overall diagnostic process is shown in Fig. 2).

Based on the patient’s clinical characteristics, pathological findings, and sequencing results, she was diagnosed with WD and MODY. She was immediately advised to consume a low-copper, diabetic diet and was administered d-penicillamine and insulin.^[6]^ Approximately 40 days after the administration of d-penicillamine, the patient’s urinary copper level rose to 1536.4 µg/24 hours, a figure that aligns with the expected increase in urinary copper excretion seen in patients with WD, thereby corroborating the diagnosis.

## 3. Discussion

WD is an uncommon hereditary disorder caused by mutations in the *ATP7B* gene encoding a copper-transporting P-type ATPase. WD is characterized by Cu accumulation in different organs, causing gradual degradation.^[7]^ Excessive Cu^2+^ in hepatocytes leads to the production of reactive oxygen species, according to Haber–Weiss reaction and the subsequent Fenton reaction. This process is called copper-mediated cuproptosis and regarded as a new way of cell death. Also, in patients with WD, iron deposits increased, which results in ferroptosis.^[8]^

More than 300 *ATP7B* mutations have been associated with WD.^[9]^ Typical clinical manifestations of WD include low ceruloplasmin and serum copper levels, although a few patients with normal ceruloplasmin and copper levels have been reported. The hepatic features of WD range from absence of symptoms to hepatic failure. A previous study reported that 40/57 children with WD were asymptomatic.^[10]^ Liver cirrhosis is commonly reported in young patients with WD, as in this report. As liver cirrhosis can be life-threatening, early diagnosis is crucial. However, the diagnosis of WD is challenging because some patients are asymptomatic or have nonspecific symptoms. In addition, the laboratory investigations used to diagnose WD, such as serum ceruloplasmin levels, may be unreliable. Approximately one-third of patients with WD have normal ceruloplasmin levels.^[11]^ Cp, an acute-phase response protein, increases during infections, inflammation, rheumatoid arthritis, cancer, and other conditions. Cp levels can also be increased by estrogen, pregnancy, and contraceptive pills.^[2]^ Serum ceruloplasmin levels are significantly reduced in patients with WD with nervous system damage compared to asymptomatic patients.^[12]^ After diagnosis, ophthalmologic examination showed Kayser–Fleischer corneal rings, suggesting that the presence of Kayser–Fleischer corneal rings is highly indicative of WD.^[12]^

The patient in this report was diagnosed with WD based on gene sequencing of *ATP7B*. The c.2333G>T mutation detected in *ATP7B* has been reported to be a predominant mutation in Chinese patients with WD.^[13]^ This mutation causes subcellular localization errors and transport instability in *ATP7B*. Functional studies in yeast have shown that this mutation severely impairs copper transport, leading to reduced copper excretion and weakening of the protective effect of wild-type proteins in cells exposed to excess copper.^[14,15]^ A c.3209C>G mutation was recently identified by Wu’s team.^[16]^ The frequency of this mutation was 0.08% among 632 patients with WD, and no mutations were detected in 503 healthy controls. This mutation was classified as a “likely pathogenic variant” according to ACMG Standards and Guidelines.^[17]^ The pathogenicity and function of c.3209C>G require further investigation.

The genotype–phenotype correlation of WD is heterogeneous. In addition, other factors may also affect phenotypes. Further investigation is required to elucidate the genotype–phenotype correlations in patients with WD, considering the diversity of clinical manifestations and the regional variability of *ATP7B* gene mutations. The phenotype of this patient may have been due to a compound heterozygous mutation (c.2333G>T, p. Arg778Leu, and c.3209C>G, p. Pro1070Arg), which has not been previously reported.

MODY is a rare disease caused by a single gene mutation and is often classified as type 1 or type 2 diabetes. *KLF11* gene mutation has been implicated in the pathogenesis of MODY type 7. The patient’s father had diabetes, which was consistent with the characteristics of MODY. MODY type 7 diabetes is a rare form of monogenic diabetes.^[18]^ However, the correlation between MODY and WD has not yet been fully studied.

## 4. Conclusion

In conclusion, WD diagnosis can be challenging. Hepatologists, neurologists, pediatricians, ophthalmologists, and other healthcare providers should be aware of the signs and symptoms of this disease. Pathological and *ATP7B* mutation analyses should be considered for difficult cases.

## Author contributions

**Conceptualization:** Zhen Yang.

**Data curation:** Meihong Han.

**Formal analysis:** Meihong Han.

**Investigation:** Meihong Han.

**Resources:** Meihong Han.

**Visualization:** Meihong Han.

**Writing – original draft:** Meihong Han.

**Writing – review & editing:** Zhen Yang.
